# Spectroscopic and Thermal Characterization of Extra Virgin Olive Oil Adulterated with Edible Oils

**DOI:** 10.3390/foods11091304

**Published:** 2022-04-29

**Authors:** Emigdio Chavez-Angel, Blanca Puertas, Martin Kreuzer, Robert Soliva Fortuny, Ryan C. Ng, Alejandro Castro-Alvarez, Clivia M. Sotomayor Torres

**Affiliations:** 1 Catalan Institute of Nanoscience and Nanotechnology (ICN2), CSIC and BIST, Campus UAB, Bellaterra, 08193 Barcelona, Spain; ryan.ng@icn2.cat (R.C.N.); clivia.sotomayor@icn2.cat (C.M.S.T.); 2 Departamento de Calidad, Döehler Fraga, Member of Döehler Group, Collidors S/N, 22520 Fraga, Spain; blanca.puertaspujadas@doehler.com; 3 ALBA Synchrotron Light Source Experiment Division—MIRAS Beamline Cerdanyola del Valles, 08290 Barcelona, Spain; mkreuzer@cells.es; 4 Agrotecnio-CeRCA Center, Department of Food Technology, University of Lleida, 25198 Lleida, Spain; robert.soliva@udl.cat; 5 Centro de Excelencia en Medicina Traslacional, Laboratorio de Bioproductos Farmacéuticos y Cosméticos, Facultad de Medicina, Universidad de La Frontera, Av. Francisco Salazar 01145, Temuco 4780000, Chile; 6 ICREA, Pg. Lluís Companys 23, 08010 Barcelona, Spain

**Keywords:** edible oils, Raman, photoluminescence, FTIR, thermal conductivity, PCA, 2DCOS

## Abstract

The substitution of extra virgin olive oil with other edible oils is the primary method for fraud in the olive-oil industry. Developing inexpensive analytical methods for confirming the quality and authenticity of olive oils is a major strategy towards combatting food fraud. Current methods used to detect such adulterations require complicated time- and resource-intensive preparation steps. In this work, a comparative study incorporating Raman and infrared spectroscopies, photoluminescence, and thermal-conductivity measurements of different sets of adulterated olive oils is presented. The potential of each characterization technique to detect traces of adulteration in extra virgin olive oils is evaluated. Concentrations of adulterant on the order of 5% can be detected in the Raman, infrared, and photoluminescence spectra. Small changes in thermal conductivity were also found for varying amounts of adulterants. While each of these techniques may individually be unable to identify impurity adulterants, the combination of these techniques together provides a holistic approach to validate the purity and authenticity of olive oils.

## 1. Introduction

Olive oil is considered to be one of the best edible oils and an essential component in the Mediterranean diet due to extraordinary organoleptic qualities and a large number of health benefits. According to the *Codex alimentarius* of the Food and Agriculture Organization of the United Nations (FAO) [1], olive oils are classified in three categories: virgin olive, refined olive, and refined olive-pomace oils. These, in turn, are divided into different grades depending on their organoleptic qualities, median defects, and color, among other attributes. From the hierarchy list of grades among these categories, extra virgin olive oil (EVOO) is considered to have the highest nutritional value with various health benefits. Among its nutritional properties, EVOO possesses high antioxidant activity [2,3], exhibits anti-inflammatory effects [3,4], improves the metabolism of carbohydrates in patients with type-2 diabetes [5,6,7,8], reduces blood pressure and the risk of hypertension [7,9], and improves vasodilation [10,11], to name a few. These many health benefits have boosted the popularity of olive oil in recent decades [12], although this popularity has also brought about other problems associated with the adulteration and/or deliberate mislabeling of EVOO [13,14]. One of the principal motivations for olive-oil fraud is the large price gap between EVOO and other non-olive oils or even between EVOO and other types of olive oils. Due to its relative scarcity and high production/selling price, unscrupulous processors have been fined for adulterating EVOO with large amounts of cheaper oils. EVOO itself is a vegetal fat with high levels of monounsaturated fatty acids (e.g., 78%) and low levels of saturated acids (e.g., 14%), in contrast to cheap seed oils (e.g., sunflower, corn, and soybean), which have high levels of polyunsaturated fats [1]. Consequently, adulteration with other oils results in the loss of many of the healthy properties of EVOO.

There is a long list of properties that can be tested to ensure the quality of EVOO [15,16,17]. The standard and official methods to characterize EVOO are gas chromatography (GC) and high-performance liquid-chromatography (HPLC). GC is mainly used to determine the composition of the saponifiable fraction, which contains fatty acids and their derivatives, as well as the unsaponifiable fraction, which contains waxes, aliphatic alcohols, tocopherols, and phenolic compounds, among others. On the other hand, HPLC is mainly used to determine the structure of triglycerides, the quantity of pigments such as chlorophylls and carotenes, and other quality parameters (other than purity). Apart from these official methods, there are a number of alternative and complementary methods that have been suggested over the past decade. Among them, infrared and Raman spectroscopy are gaining attention [18,19,20,21,22].

This work aims at evaluating the ability to detect traces of adulteration in EVOO with three spectroscopic techniques: Raman, photoluminescence (PL), and Fourier-transform infrared (FTIR) spectroscopies. In addition, we explore the use of thermal conductivity as a potential new parameter to be used as a detection tool. Despite its relative measurement simplicity, thermal conductivity has, to date, been overlooked as a figure of merit to determine food purity. The combination of all of these techniques provides an easy method, free of sample pre-processing, to ascertain the quality and authenticity of food.

## 2. Materials and Methods

Twenty samples of EVOO were intentionally adulterated using five different types of edible oils: sunflower (La Masia, “masiasol”, Sevilla, Spain), high oleic (HO) sunflower (Carrefour, “Aceite refinado de girasol”, Madrid, Spain), 95–5% soybean–nut blend (La española, “Soy plus”, Jaen, Spain), corn (Coosol, “Maiz”, Jaen, Spain), and olive-pomace (Carrefour, “Aceite refinado de orujo de oliva”, Madrid, Spain), in volume concentrations of 5%, 10%, 20%, and 50%. A single type of EVOO (Salvatge “Les Garrigues”, Lleida, Spain) was adulterated, and the oil was provided directly from the factory to guarantee its purity. All samples were stored in a dry place protected from light to preserve their quality (see Figure S1a, Supplementary Materials).

The Raman and photoluminescence spectra were recorded using the same equipment (a T64000 Raman spectrometer using a liquid-nitrogen-cooled Symphony CCD manufactured by HORIBA Jobin Yvon, Chilly-Mazarin, France) optimized in the visible regime (400–800 nm). It was used in single-grating mode with 2400 and 300 lines per mm and a spectral resolution of at least 0.4 cm^−1^ and 0.2 nm for Raman and photoluminescence, respectively. The use of 2400 lines for Raman measurements provides a very high frequency resolution at the cost of a small frequency window. On the other hand, 300 lines allow for a larger spectroscopic window which is ideal for the broad PL signal. The measurements were performed by focusing a diode laser (532 nm) onto a transparent quartz cuvette with a 10× long working distance microscope objective (see Figure S1b,c, Supplementary Materials). The power of the laser was kept as low as possible (<0.5 mW) to avoid any possible damage from self heating of the samples. For the photoluminescence measurements (also known as fluorescence spectroscopy), all samples were measured using 3 accumulations with the same integration time of 0.3 s with a fixed focal plane, to allow for direct comparison between each sample. For each sample, 5 to 10 spectra were recorded at positions on the sample.

FTIR spectra were recorded (64 co-added scans) by a Hyperion 3000 infrared (IR) microscope coupled to a Vertex 70 spectrometer manufactured by Bruker (Billerica, MA, USA) at the infrared beamline MIRAS of the ALBA synchrotron [23]. Data was recorded with a liquid-nitrogen-cooled MCT detector. A 2–5 µL drop of oil was placed on the center of a piece of ZnSe glass and pressured with a second slide to create a homogenous oil film. The setup was used in the transmission configuration with a spectral resolution of 4 cm^−1^ with a Globar as the infrared light source. The IR light was focused onto the ZnSe slide using a 30× Schwarzschild condenser and collected with a matching objective.

Principal component analysis (PCA) was used to treat the FTIR spectra using the software Orange Data Mining [24]. For each sample, 50 spectra at different sample positions were recorded and concatenated in a large matrix, as displayed in Table S1 in the supplementary information. Prior to the calculation, baseline corrections, spectral normalization, and Savitzky–Golay filters (for smoothing) and derivatives (to reduce scatter effects), were applied to process the spectra (see Figure S2, Supplementary Materials).

The thermal conductivity (*k*) was determined by the bidirectional three-omega (3ω) method [25,26] over the temperature range T = 298–400 K. The bidirectional 3ω method is based on the measurement of a rise in temperature that is produced by an alternating current (AC) passing through a metallic strip via the Joule heating effect. The metal line is composed of four rectangular pads connected by pins to a narrow wire that is used simultaneously as both a heater and temperature sensor (see Figure S1d, Supplementary Materials). The two outer pads are used to apply the AC current while the inner pads are used to measure the third component voltage (3ω-voltage), which contains the information regarding the temperature rise Δ*T*. Metal heaters (Cr:Au, 5:95 nm) were deposited by physical vapor deposition onto quartz substrates (5 × 5 × 0.5 mm^3^). For the measurement, a drop of oil (~10 µL) was placed on top of the 3ω heater. First, an empty 3ω cell was measured (reference). Then, a second measurement took place using the same cell after the sample to be studied was placed on top of the heater. Assuming that heat transfer occurs only across sample-heater-substrate interfaces, the total measured temperature change of the heater (Δ*T_Total_*) is given by [25]:

(1) $$\frac{1}{\Delta T_{Total}}=\frac{1}{\Delta T_{Sample}}+\frac{1}{\Delta T_{Substrate}}$$

where Δ*T_Sample_* and Δ*T_Sustrate_* correspond to the temperature fluctuations induced by the sample (oil) and substrate (reference) located at the top and the bottom of the heater, respectively. Lubner et al. [26] showed that the error associated with this interface assumption (Equation (1)) can be as small as 1% if three experimental conditions are fulfilled: (i) the ratio of the thermal diffusivities of the sample (*α*_oil_) and the substrate (α_Sub_), α_oil_/α_Sub_ > 10^−1^; (ii) the ratio of the thermal conductivities is in the range 10^−2^ < *k*_oil_/*k*_Substrate_ < 1; and (iii) the excitation frequencies are <100 Hz (low frequency limit). In our case, the room-temperature thermal diffusivity of the oils fluctuated within the range of (0.5–0.8) × 10^−7^ m^2^ s^−1^ [27,28], while that of the quartz fluctuated within the range of (0.8–1) × 10^−7^ m^2^ s^−1^ [29], i.e., 1 > α_oil_/α_Sub_ > 0.5. The *k* of quartz is ~1.2–1.4 W m^−1^ K^−1^ [29,30], and the *k* of oils was ~0.15–0.17 W m^−1^ K^−1^ [31,32], i.e., *k_oil_*/*k_Substrat_*_e_ < 1. The frequency range used here was (5–100) Hz, which falls within the low frequency limit.

The relationship between the temperature rise and the heat generation rate can be expressed as [33,34]:

(2) $$\Delta T=\frac{P}{l\pi k}\int_{0}^{\infty }\frac{\sin^{2}\left(xb\right)}{{\left(xb\right)}^{2}\sqrt{x^{2}+iq^{2}}}dx$$

(3) $$q=\sqrt{4\pi f/\alpha }=\sqrt{4\pi fC_{V}/k}$$

(4) $$\Delta T=\Delta T_{X}+i\Delta T_{Y}$$

where Δ*T* is the complex temperature rise oscillation; *b* and *l* are the heater’s half width (5 µm) and length (1 mm), respectively; *q* is the inverse of thermal penetration depth; *C_V_* is the volumetric heat capacity; $i=\sqrt{-1}$ is the imaginary number; *f* is the excitation frequency; and *P* is the AC power. The real and the imaginary parts are proportional to the in-phase (‘X’) and quadrature (‘Y’) components of three-omega voltage.

Finally, the thermal conductivity of the oils was found by least square fitting of the in-phase signal using *k* and *C_V_* as fitting variables. A detailed description of the bidirectional technique and the full development of the equations can be found in the supporting information of our previous works [35,36].

## 3. Results

### 3.1. Photoluminescence

**Figure 1:**
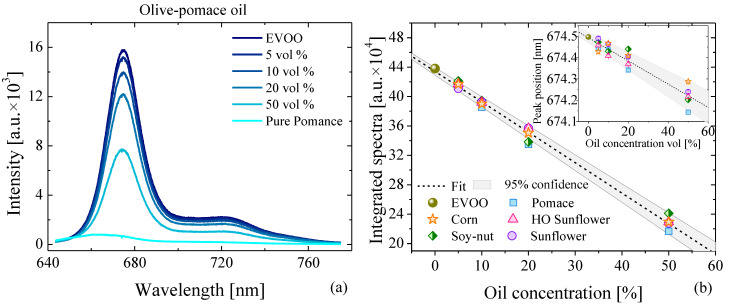
(**a**) Photoluminescence spectra of EVOO adulterated with different concentrations of olive-pomace oil. (**b**) Integrated photoluminescence spectra of the different oil mixtures as a function of the adulterant-oil concentration. The light-grey shaded region represents the 95% range of confidence region around the best-fit line. (Inset) peak position of the PL spectra as a function of adulterant-oil concentration.

The photoluminescence (PL) spectra acquired from pure and adulterated EVOO with different amounts of olive-pomace oil adulterant is depicted in Figure 1a. PL spectra for all of the different adulterated EVOO samples that were adulterated with different oils are depicted in Figure S3a–e, Supplementary Materials. Pure adulterant oils (HO sunflower, sunflower, corn, and soy–nut) do not present any PL activity under this 532 nm excitation. In the case of pure olive-pomace oil, which is a common adulterant oil, the PL is very weak with a clear blue shift in its PL peak relative to that of pure EVOO. Despite the fact that both EVOO and olive-pomace oil are derived from olives, their PL spectra present large differences from one another due to the low concentration of compounds with luminescent properties, such as pigments (e.g., chlorophyll, carotenes, and derivatives), phenols, and tocopherols in the adulterant oils [37]. For EVOO, the strong luminescence around 670 nm and 720 nm is mainly associated to the photosystem of chlorophyll [38]. The first peak is attributed to photosystem I (PSI) and the second peak is due to the combination of photosystems I and II [38]. The strong photoluminescence can be seen even by naked eye (Figure S1b,c, Supplementary Materials).

The numerically integrated PL intensity of all of the spectra as a function of the adulterant-oil concentration of different adulterant oils is shown in Figure 1b. The light-grey shaded region represents the 95% range of confidence region around the best-fit line. We note that the best-fit line passes through pure EVOO, but a clear linear decrease and variation in the integrated intensity is observed due to the negligible luminescent activity of the adulterant oils. Additionally, we observe a small blue shift of the chlorophyll/pheophytin peak as the concentration of adulterant oil increases (see inset Figure 1b). The origin of this blue shift is even more pronounced when comparing the PL spectra of pure EVOO with pure adulterant oil, since the PL peak in pure adulterant oil sits at lower wavelengths (see Figure S3, Supplementary Materials).

### 3.2. Raman Spectroscopy

**Figure 2:**
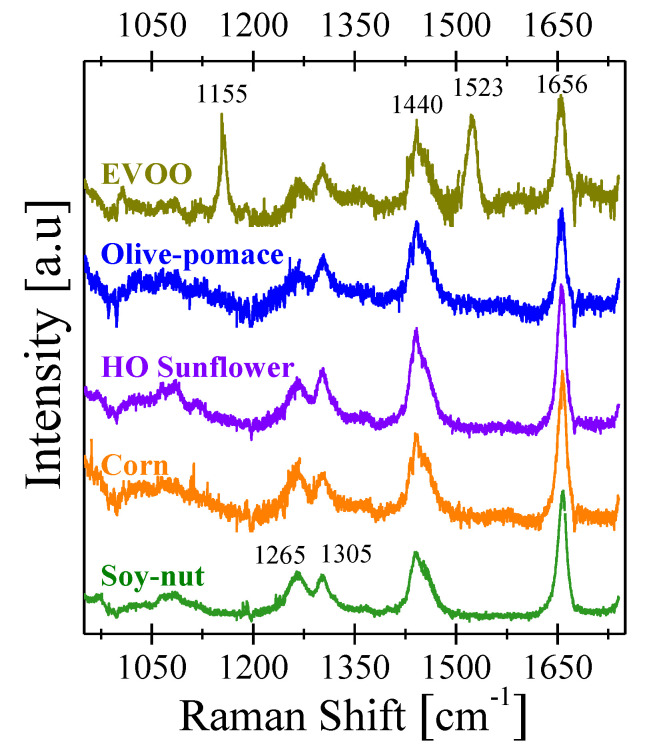
Raman spectrum of pure EVOO, olive-pomace, sunflower, corn, and soy–nut oils.

The Raman spectra of pure EVOO, olive-pomace, HO sunflower, corn, and soy–nut blend oils are depicted in Figure 2. Four common bands can be observed in all of the oils, located at ~1265, 1305, 1440 and 1656 cm^−1^, which correspond to the common Raman modes of unsaturated fatty acids such as: oleic (OA, C18:1), linoleic (LA, C18:2), and linolenic (ALA, C18:3) acid [39]. These molecules are all 18-carbon carboxylic acids with one, two, and three *cis*-double bonds, respectively. Each of the oils under study has a comparable fatty-acid composition (see Table S2 in the supplementary information), which leads to these common carboxylic acid peaks in their Raman spectra. These characteristic Raman bands have already been previously studied by El-Abassy et al. and Lv et al. [39,40]. The attribution of each of the observed peaks to their associated vibrational mode for all spectra in Figure 2 is summarized in Table 1. The remaining two Raman bands located at ~1155 and 1523 cm^−1^, which are unique to EVOO, have previously been associated with C–C and C=C stretching vibrations of the main polyene chain of carotenoids [41,42]. These additional two bands are not detected in any of the adulterant oils, including the olive-pomace oil. As was the case with the photoluminescence, Raman spectroscopy clearly distinguishes a spectroscopic difference between EVOO and all of the other edible oils, including olive-pomace oil, which shares a common derivation from olives. The absence of carotenoids in refined oils results from the degradation that they suffer during food processing, storage, and thermal treatment. Thermal treatment during the refinement process leads to the isomerization of the carotenoids and a consequent change in their molecular structure [43].

**Figure 3:**
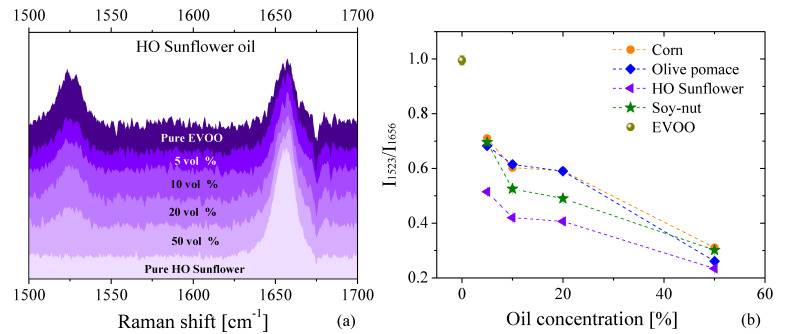
(**a**) Raman spectra of EVOO adulterated with different concentrations of high oleic sunflower oil. (**b**) Intensity ratio of the carotenoid peak (1523 cm^−1^, I_1523_) to the C=C stretching peak (1656 cm^−1^, I_1656_) as a function of the adulterant oil concentration.

Figure 3a shows the normalized Raman spectrum of pure EVOO adulterated with different amounts of HO sunflower. As the HO sunflower concentration increases, the intensity at 1523 cm^−1^ (carotenoid peak) decreases. Similar results were also found for the rest of the adulterant oils (see Figure S4, Supplementary Materials). A summary of these results are shown in Figure 3b, which presents the ratio of the numerical integration of the areas of the Raman peaks at 1523 cm^−1^ and 1656 cm^−1^. A clear decrease in I_1523_/I_1656_ ratio can be observed as the adulterant oil content increases. This effect comes from the zero Raman activity for carotenoids peaks shown by all adulterant oils studied here. Notably, the Raman spectra can be directly measured from as-packaged oil without opening and manipulating the oil, allowing for non-invasive verification even from an unopened oil bottle (see Figure S5, Supplementary Materials). Similar to the PL, the addition of adulterant oils leads to a decrease in the I_1523_/I_1656_ integral ratio. Qui et al. recently observed a similar result using the I_1523_/I_1656_ ratio to determine the free-fatty-acid (FFA) content of olive oils and found that this intensity ratio decreases linearly with FFA content, although the FFA content was obtained from the nutrition label of each of the oils [21]. Thus, the I_1523_/I_1656_ integral ratio is an additional useful figure of merit to quantify EVOO purity.

### 3.3. Fourier-Transform Infrared Spectroscopy

**Figure 4:**
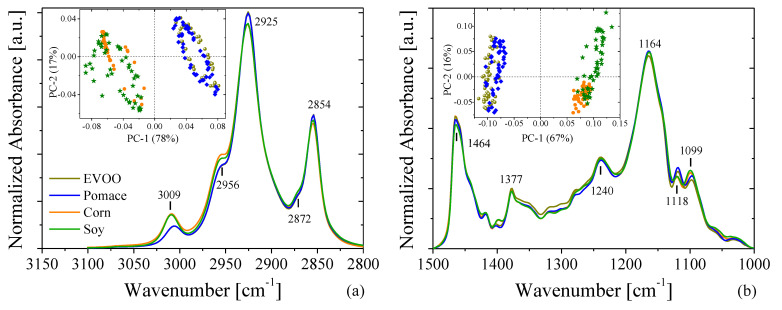
FTIR spectra of four oils: EVOO (pale green), olive-pomace (blue), corn (orange) and soy (dark green) over two different wavenumber ranges focusing on the (**a**) hydrogen stretching functional groups and (**b**) CH*_i_* (i = 1, 2, 3) functional groups present in each oil.

The IR spectra of pure EVOO, corn, soy, and olive-pomace oils are depicted in Figure 4. For ease of visualization, the spectra were separated in two wavenumber ranges: (3150–2800) cm^−1^ and (1500–1000) cm^−1^. The first window shows the characteristic IR peaks resulting from hydrogen stretching functional groups, while the second window shows other bond deformations and bending that are primarily associated with vibrations of CH*_i_* groups (with *i* = 1, 2, 3) and C–O bonds [44]. Unlike the PL and Raman results, the FTIR spectra shows remarkable similarities between the spectra of the studied samples, making them difficult to differentiate. Therefore, a deep analysis using principal component analysis (PCA), a technique that allows for patterns and variations within a dataset to be readily visualized, was performed to allow for facile differentiation of each of the spectrum from one another. PCA analysis is relatively common in food chemistry, as optical spectra tend to be very similar within particular foods and their associated derivatives. The results of this analysis are displayed in the inset of Figure 4a,b. Our results showed that EVOO and olive-pomace oils could not be differentiated from one another in FTIR spectroscopy, as the PCA scores were almost identical. However, the PCA scores of corn, soy–nut oil, and sunflower oils showed clear differences when compared with EVOO.

**Figure 5:**
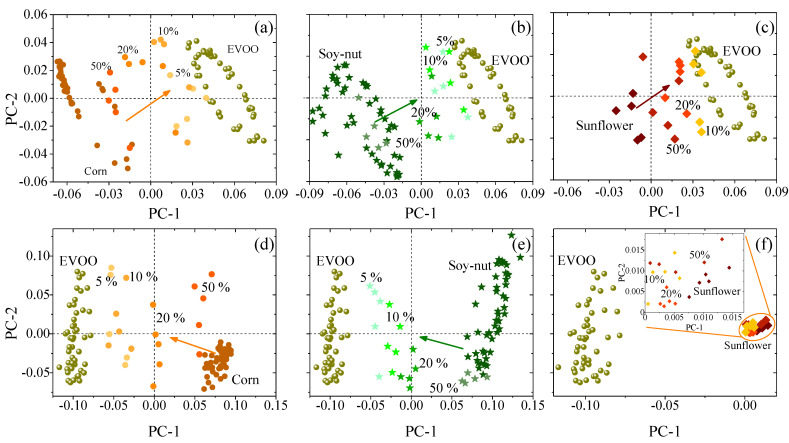
PCA score plots of oil mixtures at the 3000 cm^−1^ and 1300 cm^−1^ window: (**a**,**d**) corn–EVOO, (**b**,**e**) soy–nut–EVOO, and (**c**,**f**) sunflower–EVOO. The inset in (**f**) shows a zoom around the PCA scores of the sunflower-based sample. The color gradient in each figure indicates the evolution of the PCs from pure adulterant (darker colors) to smallest amount of adulterant (lighter colors).

At the most superficial level, a quick differentiation of the IR spectra of the oils was established via PCA, though a deeper analysis of the PCA scores of the adulterated EVOO is possible. Figure 5 shows the subsequent PCA analysis of IR spectra of adulterated EVOO. This rapid and simple PCA analysis highlights the impurities added to EVOO by showing a shift in the scores of adulterated samples as the adulterant oil increases. The shift is observed even with less than 5% of added adulterant (Figure 5a,b,d,e). Similar results have been observed by Vanstone et al. [45], who demonstrated the potential of a combination of near-infrared spectroscopy with PCA to detect EVOO adulteration at levels as low as 2.7%, given an unadulterated reference sample (i.e., pure EVOO). However, we demonstrate similar conclusions with FTIR in the mid-IR spectral range, which is advantageous as molecular fundamental vibrational modes lie in the mid-IR, while spectral measurements in the near-IR are measurements of molecular vibrational overtones. While the PCA alone exhibits potential in its ability to discriminate similar spectra, the addition of a multivariable regression model will be necessary to obtain true quantification of EVOO adulteration.

**Figure 6:**
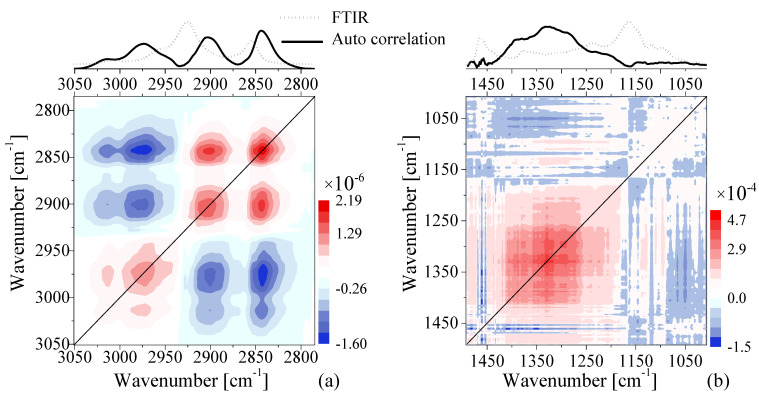
Contour map of the synchronous 2D FTIR correlation spectra of the EVOO–pomace mixtures at the (**a**) 3000 cm^−1^ and (**b**) 1300 cm^−1^ frequency windows. The spectra above the 2D plots provide the auto correlation spectrum (black solid lines) of each 2DCOS map. The average of the FTIR spectra in each window is also included for comparison (grey dashed lines).

As the PCA of FTIR spectra did not show significant differences between EVOO and olive-pomace, we applied two-dimensional correlation spectroscopy (2DCOS) to gain greater insight into the FTIR spectra. The 2DCOS technique is a mathematical method for analyzing changes in a signal produced by an external perturbation (e.g., a change in temperature, pressure, pH, concentration of mixtures, etc.). To calculate the 2DCOS map we used the concentration of olive-pomace oil as an external perturbation and the spectra dataset was ordered from pure EVOO (0% of oil adulterant) to pure pomace (100% of oil adulterant), i.e., 0, 5, 10, 20, 50, 100%. The raw spectra were baselined and normalized using the most-intense band for each frequency window in Figure 4. The average spectrum was used as a reference spectrum following the same procedure as reference [46]. The 2DCOS analysis was performed with the Mat2dcorr Matlab toolbox [47]. Figure 6 shows the synchronous 2DCOS map at the 3000 cm^−1^ (Figure 6a) and 1300 cm^−1^ (Figure 6b) FTIR frequency windows. The respective autocorrelation and FTIR-averaged spectra are shown above each frequency window. Autocorrelation spectra are defined by a diagonal line along the 2DCOS map and their bands are known as autopeaks. The autopeaks represent real changes between the FTIR spectra that are produced by the external perturbation (such as the addition of olive-pomace oil, in this case). The autocorrelation spectra show three autopeaks located at 2844, 2900, and 2974 cm^−1^ in the 3000 cm^−1^ window and only one autopeak around 1330 cm^−1^ for the 1300 cm^−1^ window. The comparison of the auto correlation and FTIR spectra show that the larger changes among the spectra occur at wavelengths where the FTIR spectra is very weak, which indicates why the PCA analysis was not able to find significant differences between the olive-pomace oil and EVOO. Furthermore, a 2DCOS analysis of pure EVOO oils was also performed using the same data treatment (Figure S6, Supplementary Materials) to verify that the observed variations are not artificial variations resulting from the data treatment such as background subtraction and/or normalization. In this analysis, the same large variation in autopeaks are in fact observed around 2844 and 2900 cm^−1^, indicating that such variations are dependent not only on real significant differences in oil concentration, but also on experimental fluctuations. Notably, autopeaks around 2974 cm^−1^ are only present in the EVOO/olive-pomace 2DCOS map. Consequently, while this type of data processing enables even such small fluctuations to be used as identifiers for authentication between oils of similar origin, additional processing and identification of 2DCOS peaks may first be required.

### 3.4. Thermal Conductivities

**Figure 7:**
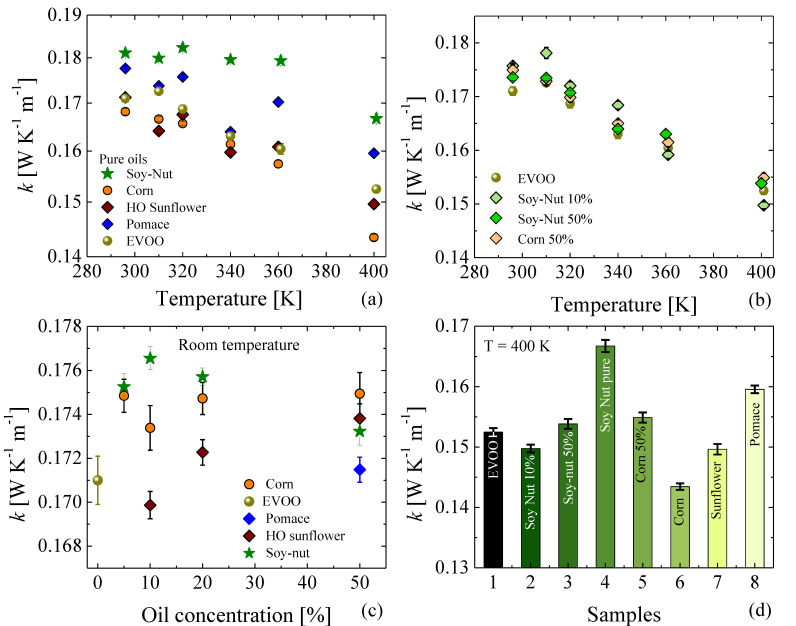
(**a**) Thermal conductivity of pure oils as a function of temperature. (**b**) Thermal conductivity of adulterated oils as a function of temperature. (**c**) Room-temperature thermal conductivity as a function of adulterant oil concentration. (**d**) Comparison of the thermal conductivity at 400 K for different pure oils or adulterated EVOO.

The temperature dependence of the thermal conductivity (*k*) of pure oils and of three adulterated mixtures are shown in Figure 7a,b, respectively. A monotonic decrease in the thermal conductivity as the temperature increases can be observed in all of the studied samples. A similar temperature dependence was also reported by Turgut et al. [31]. Interestingly, the temperature dependence of the k of the EVOO–soy–nut oil mixture follows the same temperature dependence as the pure EVOO, even at 50% of soy–nut oil concentration. A comparative analysis of the k values at room temperature (Figure 7c) and at 400 K (Figure 7d) demonstrate noticeable differences between each pure oil and between EVOO and its adulterated mixtures. This highlights the ability of the k—which, to date, has tended to be overlooked as a useful figure of merit in food authentication—to provide information that enables the distinction of pure and adulterated oils, or, more generally, other food products as well.

## 4. Discussion

In this work, the potential of four different characterization techniques to detect traces of adulteration in EVOO were analyzed. Photoluminescence, Raman, and Fourier-transform infrared spectroscopies demonstrated the successful detection of small traces of adulteration on the order of 5%, while the thermal conductivity analysis showed small but constant fluctuations as a function of adulterant oil concentration. Notably, we demonstrated four different characterization methods that are able to rapidly assess the purity of EVOO. Photoluminescence showed a linear decrease in the peak intensity and position as the adulterant oil concentration was increased due to a decrease in the amount of chlorophyll and pheophytin, which are naturally present in EVOO but absent in the adulterant oils. Raman spectroscopy also presented a clear difference between the spectra of EVOO and adulterant oils (even in olive-pomace oil, which is also derived from olives) was also found. Notably, two peaks at ~1155 cm^−1^ and 1523 cm^−1^ were detectable only in EVOO. These modes are associated with the polyene chain of the carotenoids that are naturally presented in EVOO but absent in the adulterants. A clear decrease in the intensity ratio between the peaks at 1523 cm^−1^ (only presented in EVOO) and 1656 cm^−1^ (a common mode presented in all the studied oils) was observed as a function of the adulterant-oil concentration. While a rough comparison between the IR spectra did not show appreciable differences, a statistical analysis showed grouping of the spectra and distinguished a remarkable difference in the PCA scores between pure EVOO and adulterated oils, demonstrating detection of as low as 5% adulterant concentration via FTIR spectroscopy. It is important to note that, while even PCA did not show significant differences between EVOO and EVOO–pomace mixtures, a deeper analysis using a two-dimensional correlation treatment was sensitive to small fluctuations around 2900 cm^−1^. This result is a nascent effort that demonstrates the potential of 2DCOS analysis for the detection of EVOO adulterated with oils of very similar origin. Finally, an appreciable fluctuation in the thermal conductivity of EVOO was observed for different amounts of adulterant oils. Thermal conductivity has previously been overlooked as a simple but useful figure of merit for assessing food authenticity, but is also a useful manner in which purity can be ascertained. These results highlight the potential of these techniques to detect adulteration, and indicate that the results of the current study can be used as a starting point for the development of spectroscopic methods that allow for the effective and efficient detection of adulteration in olive oils by aiding in identification and classification. While each technique independently may fail to reliably capture small amounts of adulteration in EVOO given the complexity and chemical variability in the oils, a combination of all of them together provides a more holistic base for authentication. For example, as was observed in the case of the FTIR spectra, it is difficult to differentiate EVOO from olive-pomace oils, due to their common origin, though other techniques such as Raman can clearly distinguish the two. Future subsequent development of multiple sensors incorporating and combining these techniques will allow for the acquisition of complete spectral data sets that are critical for precise EVOO authentication. Beyond the authentication of EVOO, the combination of spectroscopic and thermal techniques has the potential to facilitate simplified authentication throughout the food industry.

## Figures and Tables

**Table 1:** Assignment of the Raman bands of the edible oils.

Frequency [cm^−1^]	Vibrational Mode
1155	C–C stretching (carotenoid)
1265	=C–H bending scissoring
1305	C–H bending twisting
1440	C–H bending scissoring
1523	C=C stretching (carotenoid)
1656	C=C stretching

## Data Availability

Raw data can be provided by the corresponding author (ECA) on reasonable request.

## Supplementary Materials

The following supporting information can be downloaded at: https://www.mdpi.com/article/10.3390/foods11091304/s1, Figure S1: Sample preparation; Figure S2: IR spectra second derivative and pc loading of EVOO, pomace, corn, and soy-nut oils; Figure S3: Photoluminescence of EVOO adulterated with different concentrations of: (a) corn, (b) soy-nut blend, (c) high oleic sunflower, (d) sunflower oils, and (e) olive-pomace oils; Figure S4: Normalized Raman spectra of EVOO adulterated with different concentrations of: (a) ol-ive-pomace, (b) soy-nut blend, and (c) corn oils; Figure S5: Normalized Raman spectra of EVOO measured directly from its package; Figure S6: 2DCOS map of pure EVOO; Table S1: Schematic representation of FTIR dataset for PCA; Table S2: Acid content of the studied edible oils [48].
