# Supplementary material for: Spectroscopic and Thermal Characterization of Extra Virgin Olive Oil Adulterated with Edible Oils
Source: Foods. 2022 Apr 29;11(9):1304. doi: 10.3390/foods11091304 (PMC9100626; doi:10.3390/foods11091304)
Supplement: Supplementary file 1 [file foods-11-01304-s001.zip › foods-1705543-supplementary.pdf]

Supplementary material

Spectroscopic and thermal characterization of extra virgin olive oil adulterated with edible oils

Emigdio Chavez-Angel[1,*], Blanca Puertas[2], Martin Kreuzer[3], Robert Soliva Fortuny[4], Ryan C. Ng[1], Alejandro Castro-Alvarez[5*] and Clivia M. Sotomayor Torres[1,6]

[1]Catalan Institute of Nanoscience and Nanotechnology (ICN2), CSIC and BIST, Campus UAB, Bellaterra, 08193 Barcelona, Spain.

[2] Departamento de calidad, Döehler Fraga, member of Döehler group, Collidors S/N, E-22520 Fraga, Spain.

[3] ALBA Synchrotron Light Source Experiment Division – MIRAS Beamline Cerdanyola del Valles, 08290 Barcelona, Spain.

[4] Department of Food Technology, University of Lleida – Agrotecnio-CeRCA Center, 25198 Lleida, Spain.

[5] Laboratorio de Bioproductos Farmacéuticos y Cosméticos, Centro de Excelencia en Medicina Traslacional, Facultad de Medicina, Universidad de La Frontera, Av. Francisco Salazar 01145, Temuco 4780000, Chile.

[6] ICREA, Pg. Lluís Companys 23, 08010 Barcelona, Spain.

* Correspondence: emigdio.chavez@icn2.cat (ECA) Alejandro.castro.a@ufrontera.cl (ACA)

Sample preparation

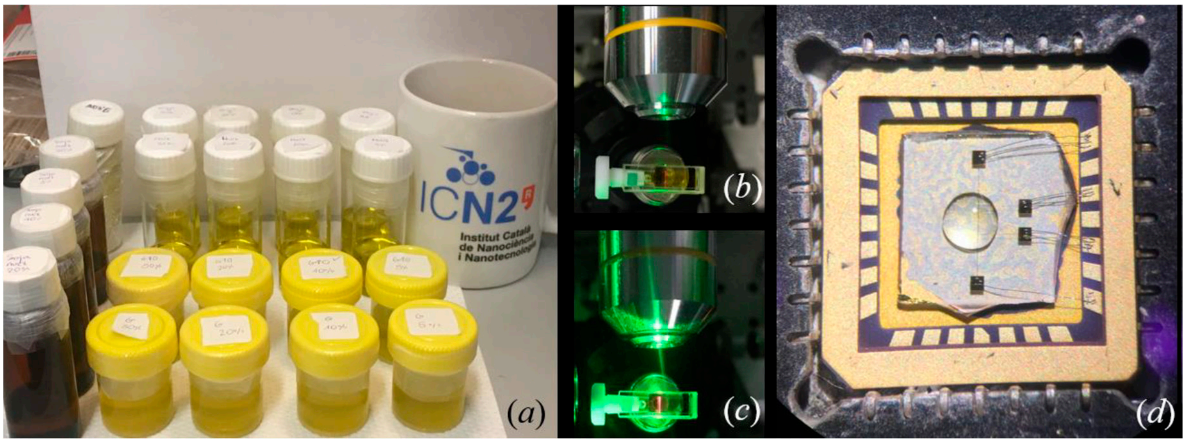

Figure S1. (a) All the samples used in this study, (b) and (c) quartz cuvette for Raman spectroscopy and photoluminescence showing the luminescence created by the green laser. (d) three-omega chip for thermal characterization.

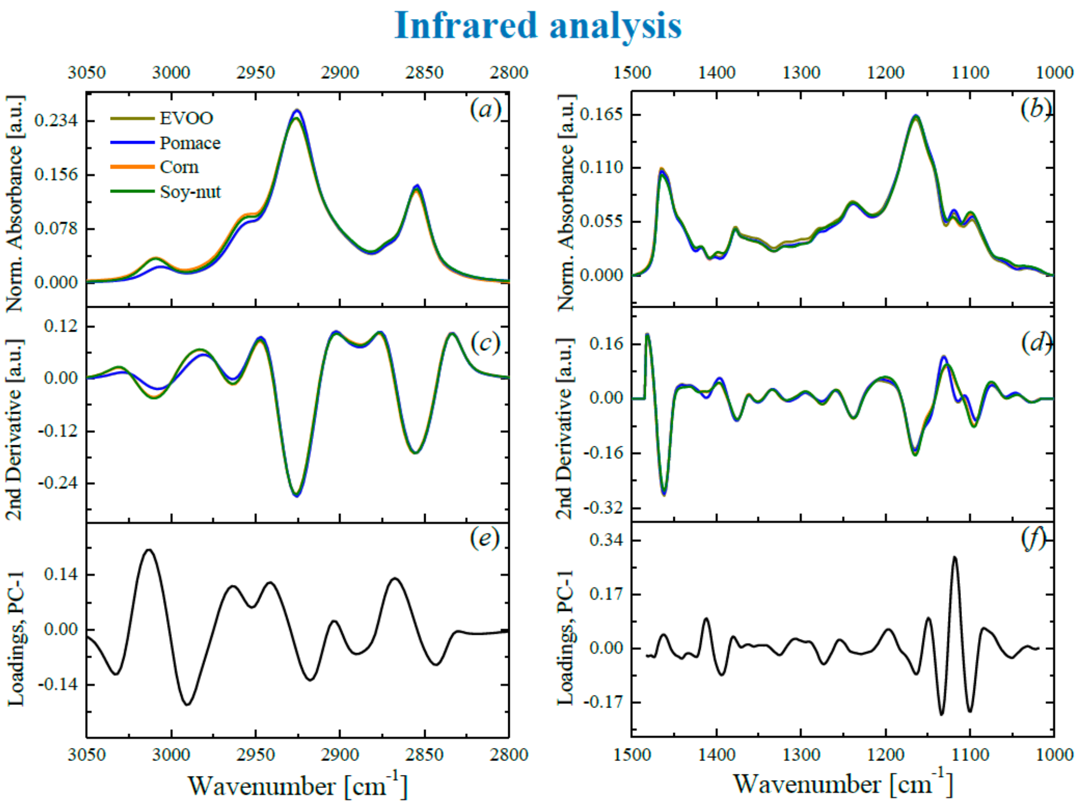

Figure S2. IR spectra second derivative and pc loading of EVOO, pomace, corn, and soy-nut oils.

Photoluminescence analysis

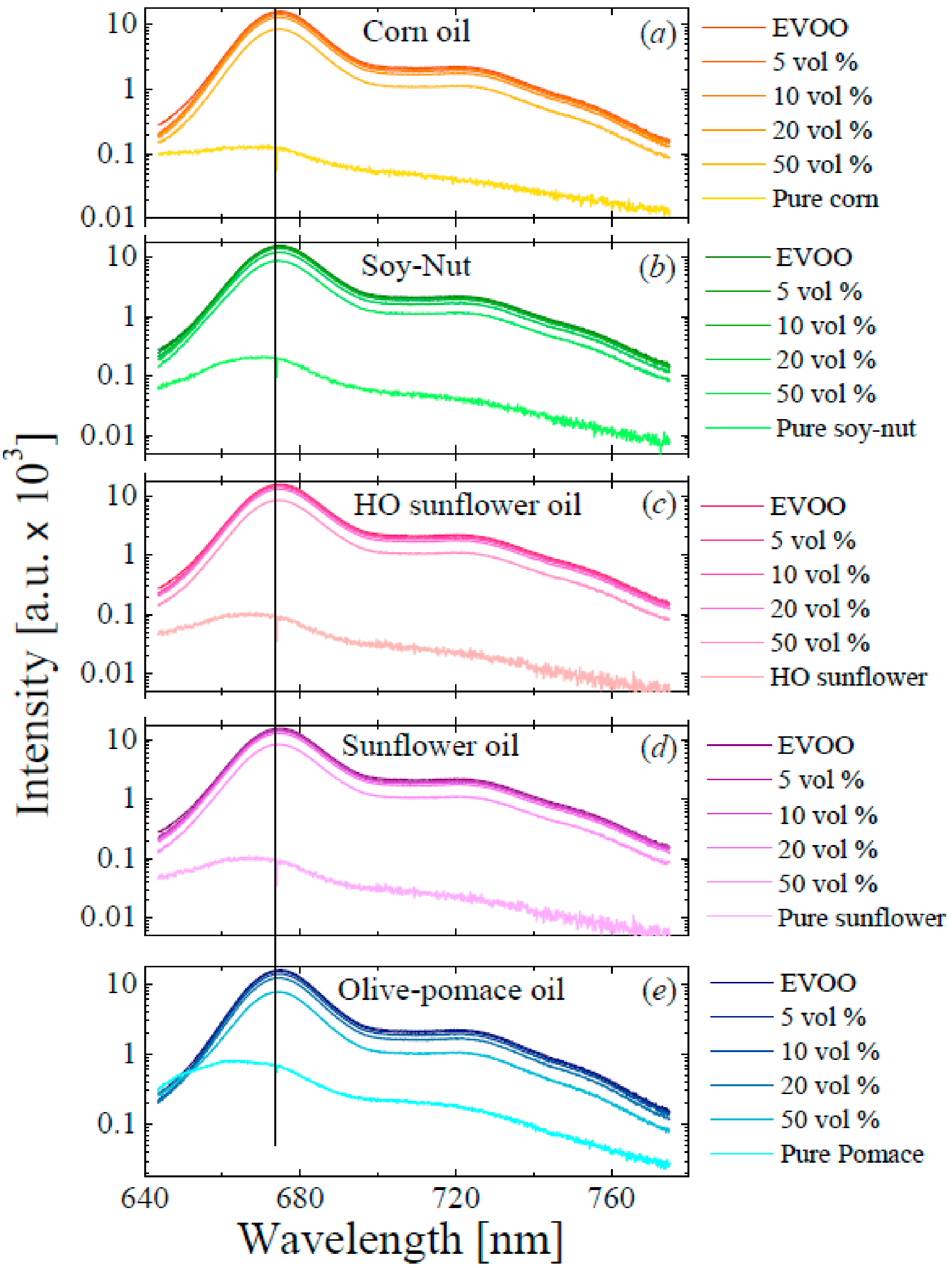

Figure S3. Photoluminescence of EVOO adulterated with different concentrations of: (a) corn, (b) soy-nut blend, (c) high oleic sunflower, (d) sunflower oils, and (e) olive-pomace oils.

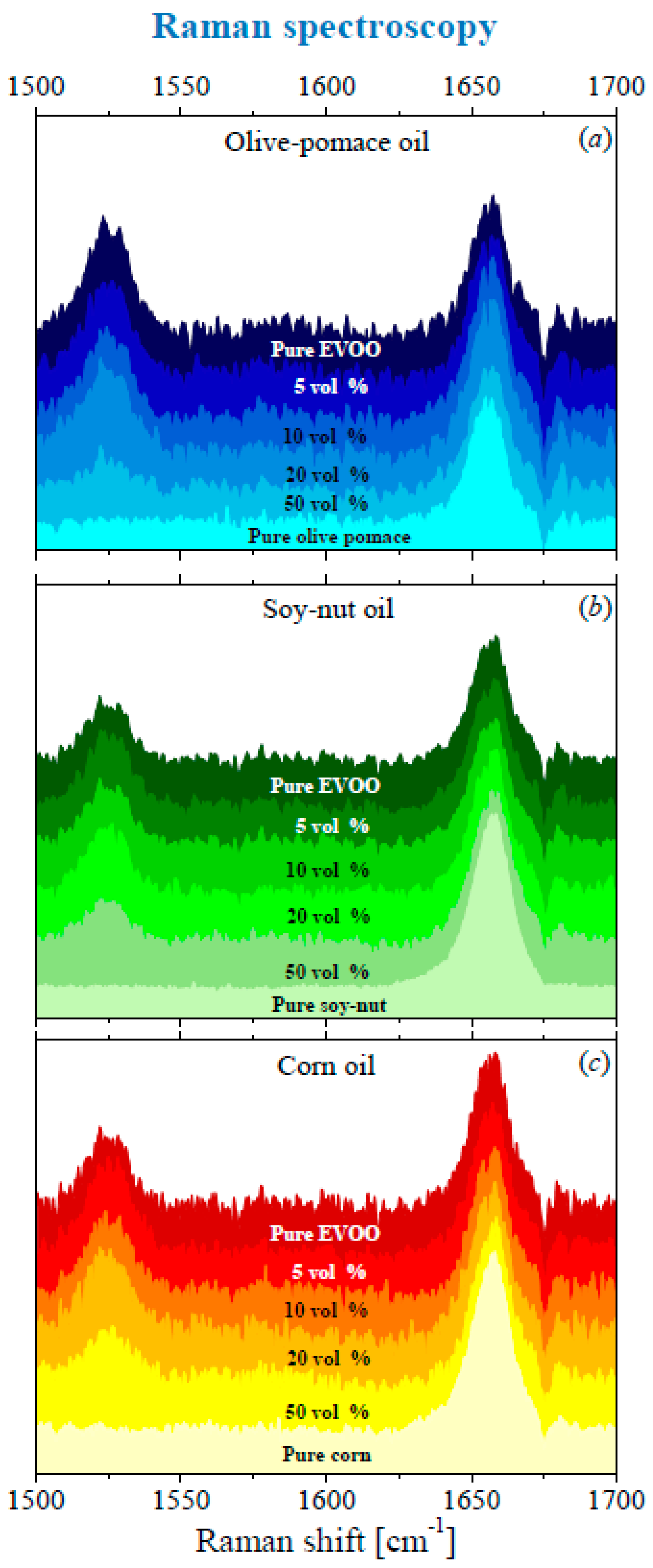

Figure S4. Normalized Raman spectra of EVOO adulterated with different concentrations of: (a) olive-pomace, (b) soy-nut blend, and (c) corn oils.

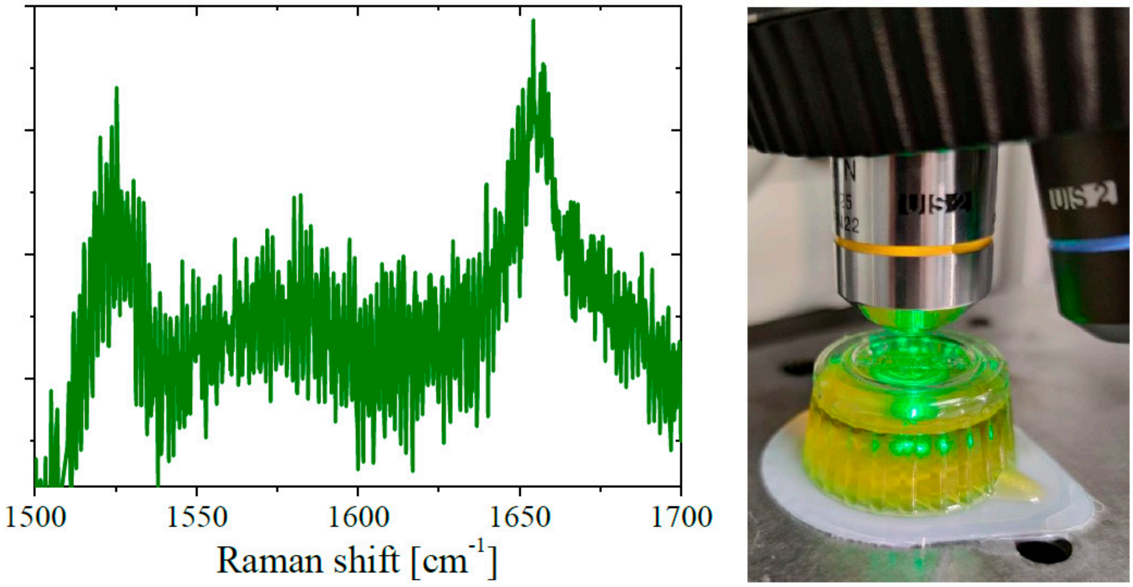

Figure S5. Normalized Raman spectra of EVOO measured directly from its package.

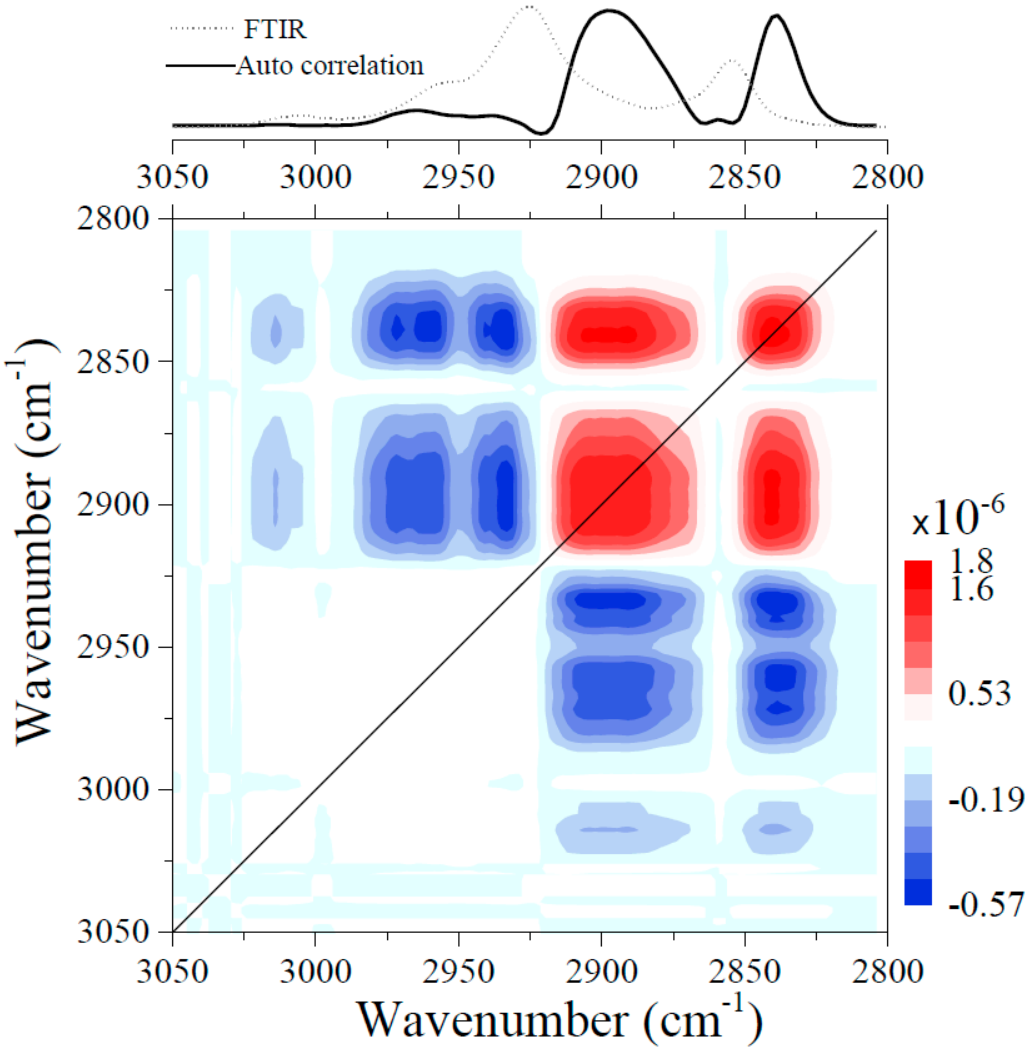

Figure S6. 2DCOS map of pure EVOO.

Table S1. schematic representation of FTIR dataset for principal component analysis.

Sample \ Wavenumber (cm^{-1})	**3048**	**3047**	**3046**	**3045**	**3044**	. . .
EVOO 1	$I_{1, 3048}$	$I_{1, 3047}$	$I_{1, 3046}$	$I_{1, 3045}$	$I_{1, 3044}$	. . .
EVOO 2	$I_{2, 3048}$	$I_{2, 3047}$	$I_{2, 3046}$	$I_{2, 3045}$	$I_{2, 3044}$	. . .
EVOO 3	$I_{3, 3048}$	$I_{3, 3047}$	$I_{3, 3046}$	$I_{3, 3045}$	$I_{3, 3044}$	. . .
⋮	⋮	⋮	⋮	⋮	⋮	
EVOO 49	$I_{49, 3048}$	$I_{49, 3047}$	$I_{49, 3046}$	$I_{49, 3045}$	$I_{49, 3044}$	. . .
EVOO 50	$I_{50, 3048}$	$I_{50, 3047}$	$I_{50, 3046}$	$I_{50, 3045}$	$I_{50, 3044}$	. . .

Table S2. Acid content of the studied edible oils [1].

Oils	Acid content			
	PA (C16:0)	OA (C18:1)	LA (C18:2)	ALA (C18:3)
Corn	8.6-16.5 %	20-42.2%	34.0-65.6 %	< 2 %
Soy	8-13.5 %	17-30 %	48-59 %	4.5-11 %
HO Sunflower	2.6-5 %	75-90.7 %	2.1-17 %	< 0.3 %
EVOO	7.5-20 %	55-83 %	3.5-21 %	< 1.5 %
Olive-Pomace	7.5-20 %	55-83 %	3.5-21 %	< 1.5 %

References

1. FAO. Codex standard for fats and oils from vegetable sources. (1999).
